# Active oxygen species adsorbed on the catalyst surface and its effect on formaldehyde oxidation over Pt/TiO_2_ catalysts at room temperature; role of the Pt valence state on this reaction?

**DOI:** 10.1039/c7ra11294g

**Published:** 2018-01-18

**Authors:** Geo Jong Kim, Sang Moon Lee, Sung Chang Hong, Sung Su Kim

**Affiliations:** Department of Environmental Energy Engineering, Graduate School of Kyonggi University 94-6 San, Iui-dong, Youngtong-ku Suwon-si Gyeonggi-do 443-760 Republic of Korea schong@kgu.ac.kr +82-31-2544941

## Abstract

Pt/TiO_2_ catalysts, prepared by reduction pretreatment, showed enhanced catalytic activities in formaldehyde oxidation. X-ray photoelectron spectroscopy analysis confirmed that catalytic activity was affected by Pt valence states in the Pt/TiO_2_ catalyst. Using O_2_ re-oxidation tests, we showed that there was a correlation between the area of oxygen consumed and the ratio of metallic Pt species on the catalyst surface. The O_2_ re-oxidation ability was involved in the production of the adsorbed formate intermediate from HCHO, confirmed through diffuse reflectance infrared Fourier transform spectroscopy analysis. Furthermore, metallic Pt species were involved in the oxidation of adsorbed CO to CO_2_.

## Abstract 2

Two catalyst factors were identified that affected the reaction rate in the room temperature oxidation of HCHO.

## Graphical abstract

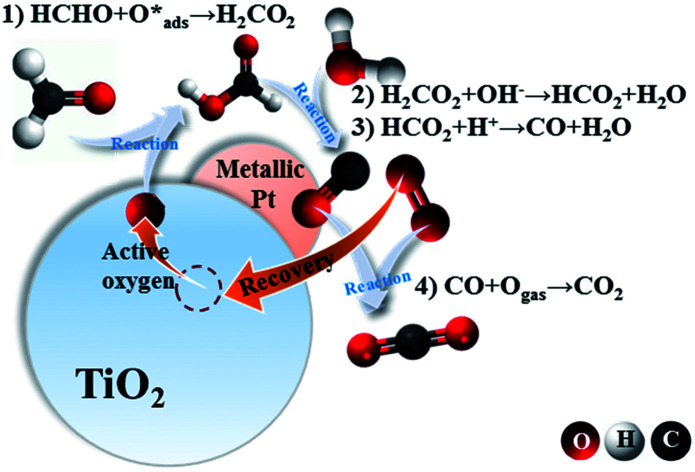

## 1 Introduction

Demand for comfortable and healthy living space has increased due to the rise in living standards. Because a significant portion of the modern lifestyle is spent indoors, the necessity of improving the air quality of living and work environments is increasing. The removal of harmful substances from indoor air has, therefore, emerged as a concern.

Indoor air quality is affected by various substances, such as carbon monoxide (CO), formaldehyde (HCHO), nitrogen dioxide (NO_2_), ozone (O_3_), particulates (PM10), asbestos, volatile organic compounds (VOCs), microbial substances, and radon (Rn).^1–3^ Among these substances, formaldehyde is most harmful to human health, and is emitted from several common sources including thermal insulation materials, furniture, tobacco smoke, deodorant, cosmetics, and plywood burning. Upon short-term exposure, formaldehyde penetrates the body and enters mucous membranes in the eyes and nose, while long-term exposure can cause diarrhea, coughing, emotional instability, skin disease (atopy), and amnesia.^4,5^

Extensive research on HCHO oxidation at room temperature has been conducted recently, with principal technologies using noble-metal-supported catalysts.^6–11^ Zhang *et al.* reported that HCHO could be completely oxidized to CO_2_ over a Pt/TiO_2_ catalyst, and they performed diffuse reflectance infrared Fourier transform spectroscopy (DRIFTS) to propose a reaction model.^12^ In this model, differences in catalytic activity were caused by the specific intermediate products produced, including CO and formate species. Nie *et al.* reported that the surface hydroxyl groups are indispensable and act synergistically of HCHO reaction, and they propose a reaction model that hydroxyl group participates in the HCHO reaction.^13^

Tang *et al.* and Kim *et al.* reported the effect of catalyst reduction as a pretreatment step on the feeding concentration of HCHO, and showed that the reduced catalysts displayed excellent activities, with an optimum reduction temperature of 873 K.^14,15^ A higher HCHO feeding concentration was also reported, resulting in drastically decreased catalytic activity, with metallic Pt species proving more active than PtO_*x*_ species. However, the report was limited to one catalyst, so further study of various supports is required.

Huang and Leung reported that Pt/TiO_2_ and Pd/TiO_2_ catalysts have strong potential for HCHO oxidation, with their activities dependent on the magnitude of the strong metal-support interaction (SMSI) phenomenon and the oxidation states of the active metals. They also reported that catalytic performance improved as the Pd or Pt oxidation states decreased, caused by the SMSI effect between the active metal and the TiO_2_ support.^16–18^ Only one TiO_2_ support was used, however, so the effect of the type of TiO_2_ support on the SMSI effect or the Pt valence was not reported. They also did not determine why metallic Pt gave such a strong HCHO conversion rate.

This study examined the activities of various Pt/TiO_2_ catalysts, using different types of supports, in HCHO oxidation at room temperature. We also performed various analyses to investigate the relationship between HCHO oxidation catalytic activity and the physicochemical character of the catalyst.

## 2 Results and discussion

### 2.1 Physicochemical characteristics of Pt/TiO_2_ catalysts and catalytic activities

8 anatase and 2 rutile type TiO_2_ supports with varying surface areas and particle sizes were chosen to investigate the influence of a catalytically active TiO_2_ support.

**Fig. 1:**
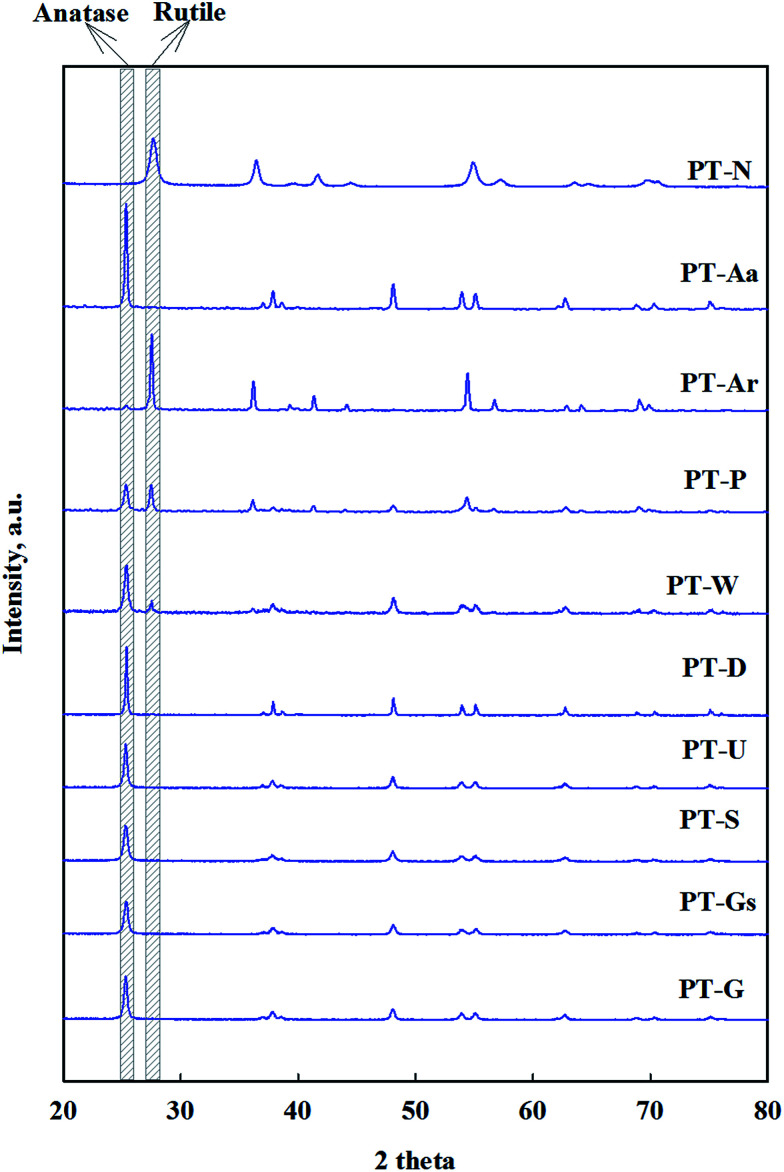
X-ray diffraction patterns of the 1% Pt/TiO_2_ catalysts.

The structural properties of Pt/TiO_2_ catalysts were investigated by XRD, shown in Fig. 1. All samples were found to exist as anatase or rutile TiO_2_ structures. It was found that Pt highly dispersed over the support, due to the absence of diffraction lines on Pt of 1% Pt/TiO_2_ catalyst, as previously reported by Zhang *et al.* and Kim *et al.*^15,19,20^

**Fig. 2:**
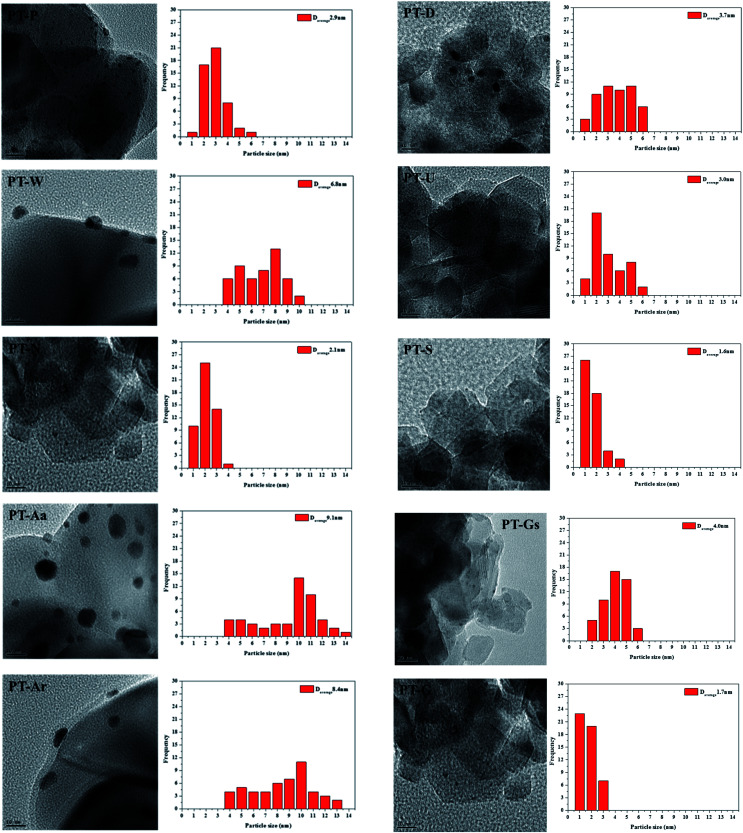
FE-TEM analysis of the 1% Pt/TiO_2_ catalysts.

The physico-chemical properties of the various TiO_2_ supports and Pt/TiO_2_ catalysts manufactured in this study are showed in Table 1. Pt particle sizes in the Pt/TiO_2_ catalysts were confirmed by TEM, shown in Fig. 2. The specific surface areas of all catalysts were relatively low, compared with raw TiO_2_, while Pt dispersion and particle size varied among the types of TiO_2_ used as supports.

**Table 1:** Physicochemical characteristics of TiO_2_ and Pt/TiO_2_ catalysts and their activities. Reaction temperature = 298 K, HCHO = 25 ppm, O_2_ = 21%, N_2_ balance, total flow = 500 ml min^−1^, SV = 360 000 h^−1^

Samples	SSA of raw TiO_2_ (m^2^ g^−1^)	SSA of Pt/TiO_2_ (m^2^ g^−1^)	*D* (%)	Reaction rate (mmol g_cat_^−1^ min^−1^)	TOF × 100 (s^−1^)
PT-Aa	11	9	2.6	4.29 × 10^−6^	0.322
PT-Ar	4	23	3.2	6.59 × 10^−6^	0.401
PT-D	76	35	3.4	8.16 × 10^−6^	0.468
PT-G	340	38	6.1	6.18 × 10^−5^	1.975
PT-Gs	250	21	6.4	1.75 × 10^−5^	0.533
PT-N	144	26	6.9	7.12 × 10^−5^	2.025
PT-P	44	30	5.9	1.75 × 10^−5^	0.578
PT-S	122	55	19.1	1.08 × 10^−4^	1.103
PT-U	149	37	13.2	9.37 × 10^−5^	1.384
PT-W	65	11	3.7	6.47 × 10^−6^	0.341

**Fig. 3:**
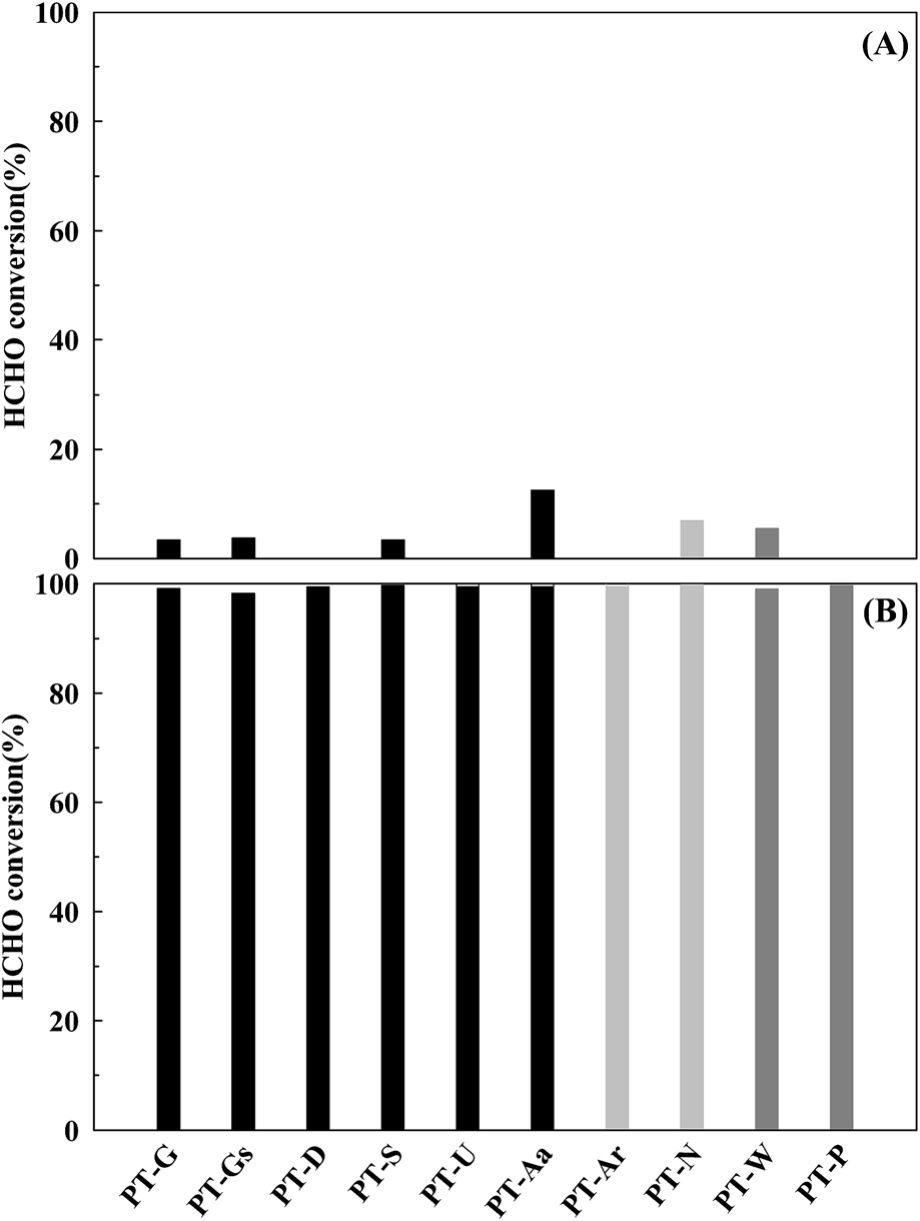
Catalytic activities of the Pt/TiO_2_ catalysts after (A) calcination only, and (B) reduction treatment. Inlet HCHO = 24 ppm, 21% O/N_2_, temperature = 298 K, SV = 60 000 h^−1^.

The Pt/TiO_2_ catalyst activities are shown in Fig. 3. The reduced catalysts (Fig. 3(B)) showed greater levels of activity than those that did not undergo the reduction pretreatment under space velocity (SV) conditions of 60 000 h^−1^. This phenomenon was thought to be caused by rich metallic Pt sites on the catalyst, it will be discussed in more detail later.^15^

Differences in activities at a SV of 60 000 h^−1^ were difficult to find. In a heterogeneous catalyst reaction, mass transfer does not influence the overall reaction rate if mass transfer is sufficiently fast, or if the catalyst reaction rate is very slow. Under these conditions, the overall reaction rate rely on the specific reaction rate of the catalyst, occurring at the active site.

**Fig. 4:**
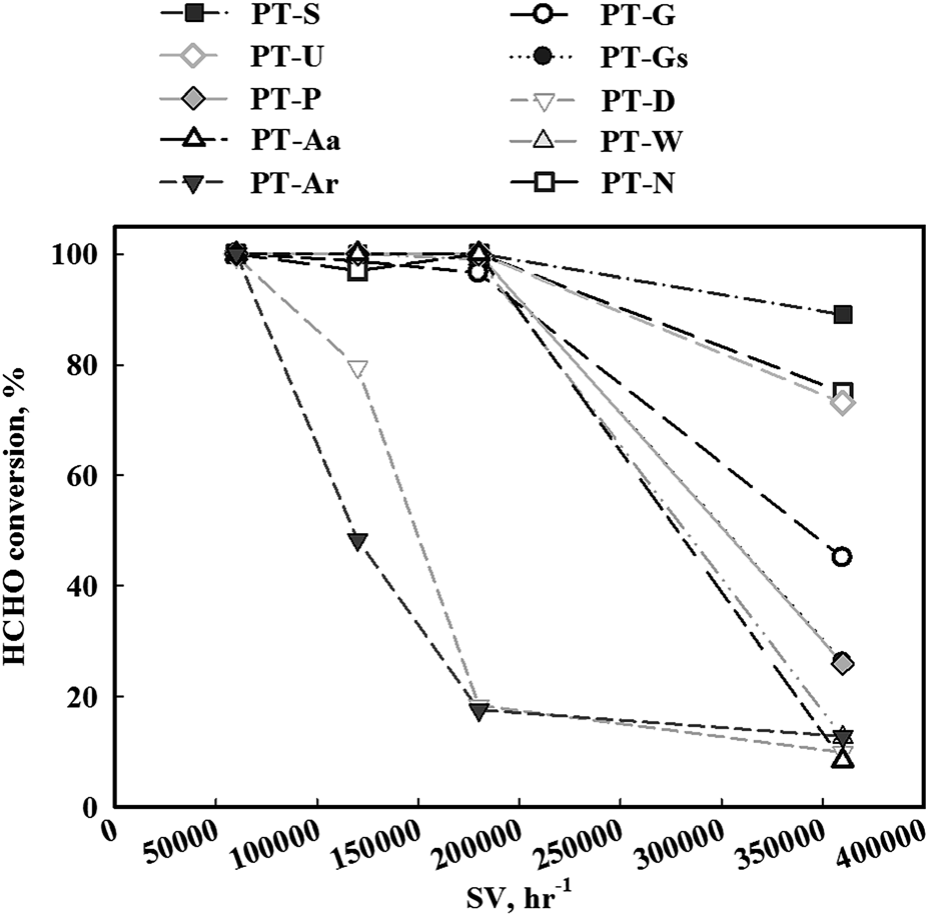
Catalytic activities of Pt/TiO_2_ catalysts after calcination and reduction treatment. Inlet HCHO = 24 ppm, 21% O/N_2_, temperature = 298 K, SV = 60 000–360 000 h^−1^.

In contrast, if the mass transfer rate is relatively slower than the catalyst reaction rate, the overall reaction rate is dependent on the mass transfer rate. In this respect, the mass transfer rate for the reduced catalyst was not sufficient, therefore, the activity test under high SV conditions (Fig. 4) was necessary. When SV was increased, the catalytic activities of all catalysts decreased, however, the magnitude of this decrease varied with the type of TiO_2_ support. The TiO_2_(S) catalyst showed the highest catalytic activity, with a HCHO conversion of 90%. The order of activity was as follows: TiO_2_(S) > TiO_2_(N) > TiO_2_(U) > TiO_2_(G) > TiO_2_(Gs) > TiO_2_(P) > TiO_2_(W) = TiO_2_(Ar) > TiO_2_(D) > TiO_2_(Aa).

The catalytic activity was also examined in terms of TOF and reaction rate, shown in Table 1. Useful information can be obtained by evaluating activity with TOF.^15,21–23^ If there was no difference between the activities regarding the TOF and reaction rate, catalyst activities would be determined by Pt dispersion, however, if the activities differed, the magnitude of activity would depend on the frequency of active sites. In this research, a difference between the two reaction activities was observed over each catalyst, suggesting that differences in catalytic activity were not due to the number of active sites, but rather differences in the intensity of activity at each catalyst active site.

### 2.2 Effect of Pt valence state and chemisorbed oxygen

Huang and Leung recently reported that the important factors of HCHO oxidation at room temperature were the valance state of the active metal and the mobility of activated chemisorbed oxygen on the catalyst surface, rather than the noble-metal-supported catalysts.^16–18^ In their reports, the mobility of chemisorbed oxygen was more important than that of lattice oxygen, and the formation of chemisorbed oxygen depended on a strong interaction between Pt and TiO_2_. This effect is known as a strong metal-support interaction (SMSI), and can be explained by changes in electron density with the movement of electrons from Ti to Pt sites by a strong interaction between a partially reduced Ti site, and Pt. Pt–O_v_–Ti^3+^ sites, which are formed between Pt and Ti, where O_v_ is an oxygen vacancy close to the Pt interfacial site.^20^ Gas-phase oxygen can dissociatively adsorb at this defect site, and the chemisorbed oxygen can then be activated at the metal-support interface, forming the highly active oxygen species involved in oxidation.^17^ Based on this, XPS analysis was carried out to investigate Pt valence and chemisorbed oxygen species in the 10 types of Pt/TiO_2_ catalyst used in this study.

**Fig. 5:**
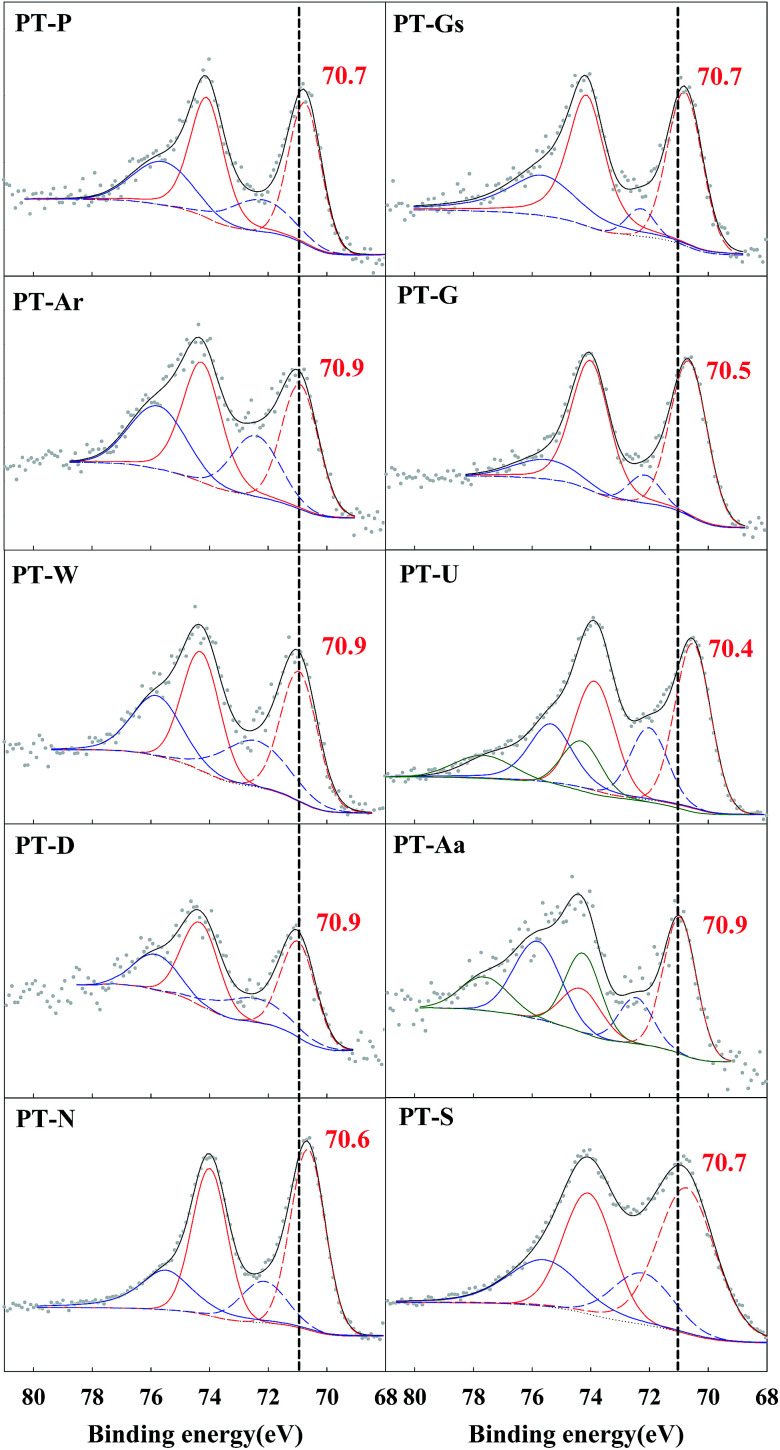
Pt 4f spectra of Pt/TiO_2_ catalysts from XPS analysis.

The positions of the Pt 4f peaks corresponding to Pt^0^, Pt^2+^ and Pt^4+^ were 70.9–71.1, 72.4–73.6 and 74.56–74.8 eV, respectively.^24,25^Fig. 5 confirms that almost all the Pt/TiO_2_ catalysts were reduced to metallic Pt (Pt^0^) or PtO (Pt^2+^). The magnitude of Pt oxidation state changes between the various catalysts. A small negative shift in binding energy was also found for metallic Pt species, which can help confirm the SMSI effect. The SMSI effect caused electrons to migrate from Ti^3+^ sites to adjacent Pt sites, resulting in enhanced electron density at the Pt–TiO_2_ interfacial site. A difference of approximately 1 eV in the binding energies of metallic Pt sites adjacent to Ti^3+^ sites was reported for the reduced Pt–TiO_2_ catalysts.^18,26^ In this study, the binding energy shift was lower than that found in the literature. This difference in the magnitude of the shift was believed to be caused by differences in catalyst preparation methods, with the use of a NaBH_4_ solution causing a more extreme SMSI effect than the use of hydrogen gas.

**Fig. 6:**
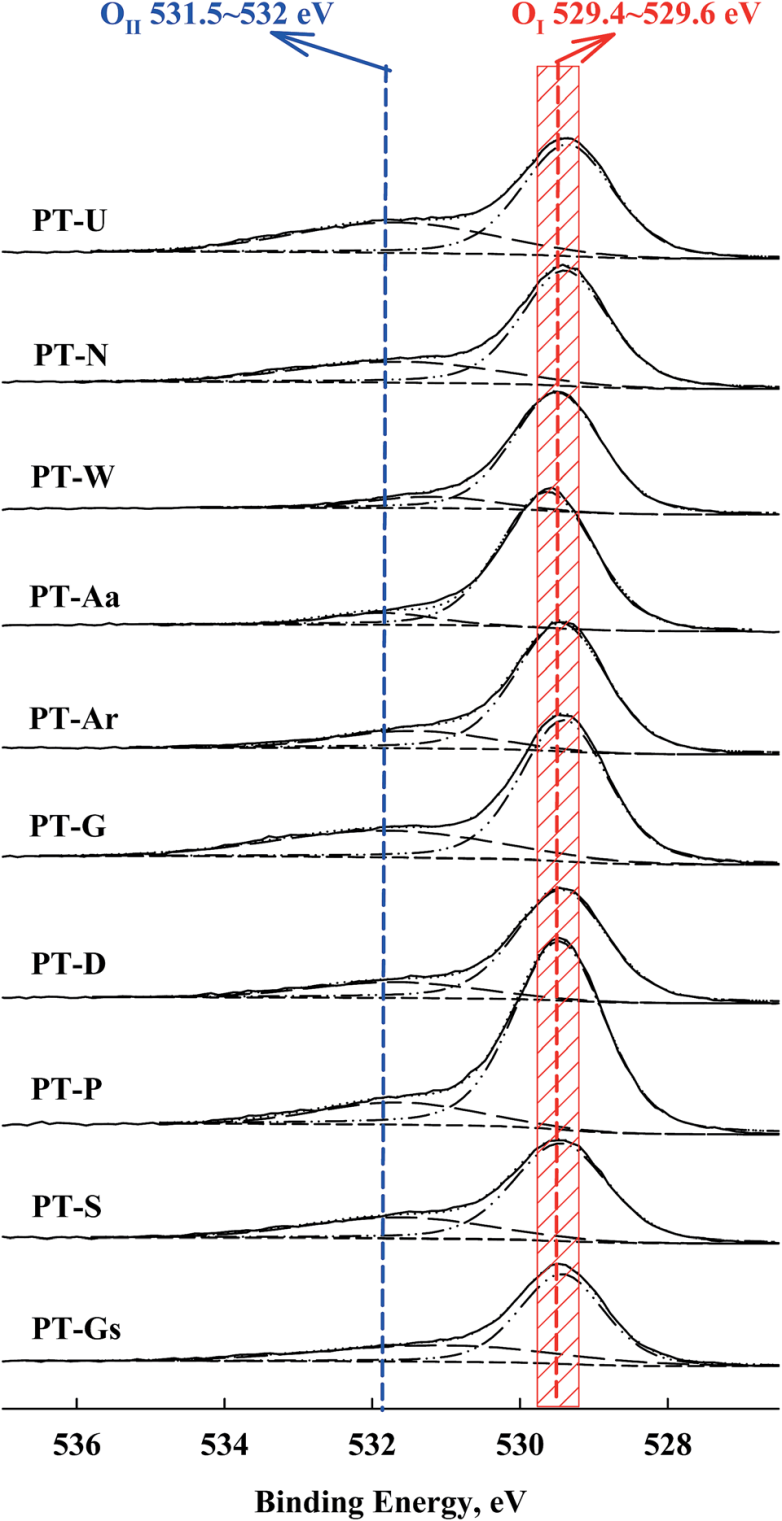
O 1s spectra of Pt/TiO_2_ catalysts from XPS analysis.

A shift in the O 1s peak was also confirmed in the XPS spectra (Fig. 6). An O 1s shoulder peak, corresponding to chemisorbed oxygen species, was observed at 531.5–532 eV in all catalysts.^27,28^ The peak positions for Pt 4f and O 1s are shown in Table 2. According to Huang and Leung, metallic Pt sites and oxygen vacancies can facilitate oxygen adsorption and activation, so the amount of chemisorbed oxygen species depended on the intensity of the SMSI effect (*i.e.* the population of metallic Pt).^18^

**Table 2:** XPS data of Pt/TiO_2_ catalysts

Catalysts	BE, eV	
	Pt 4f	O 1s, O_II_ (O_I_)
PT-Aa	70.9	531.8 (529.6)
PT-Ar	70.9	531.8 (529.4)
PT-D	70.9	531.4 (529.5)
PT-G	70.5	531.7 (529.5)
PT-Gs	70.7	531.4 (529.6)
PT-N	70.6	531.8 (529.4)
PT-P	70.7	531.8 (529.5)
PT-S	70.7	531.7 (529.5)
PT-U	70.4	531.8 (529.4)
PT-W	70.9	531.4 (529.5)

**Fig. 7:**
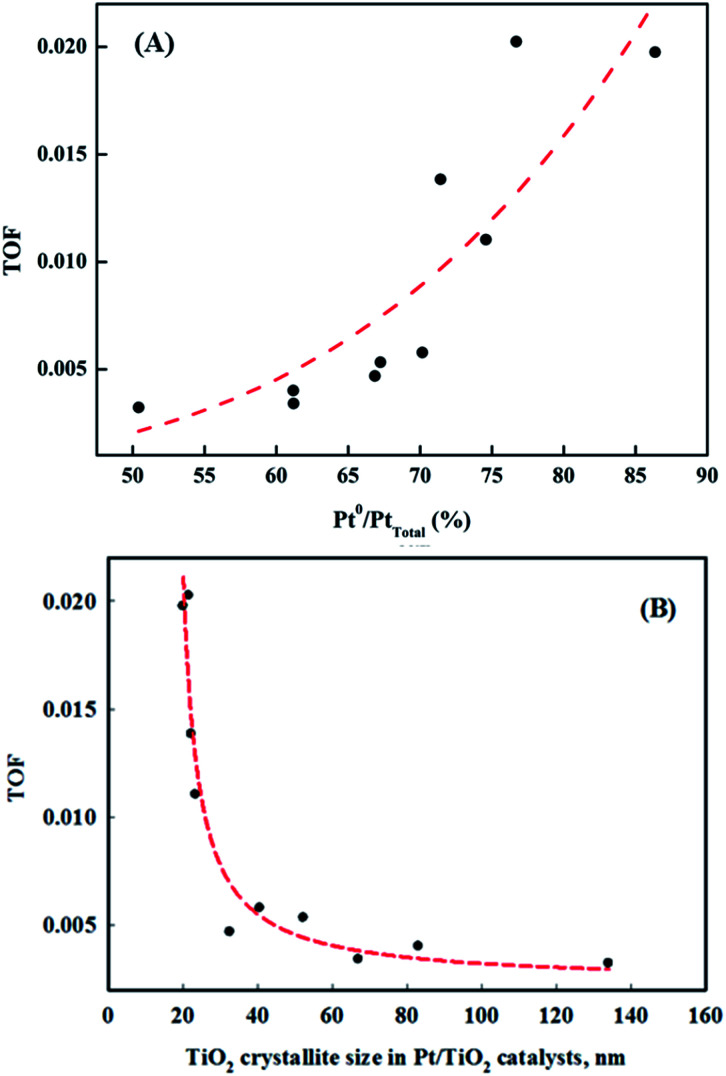
Correlation between the ratios of (A) metallic Pt/Pt_total_ and catalyst activities, and (B) metallic Pt/Pt_total_ and TiO_2_ crystallite size in the Pt/TiO_2_ catalysts.

The correlation between metallic Pt atom population and catalyst activity is shown in Fig. 7(A). A positive linear relationship between catalytic activity and the amount of reduced metallic Pt formed on the catalyst surface was confirmed. It was consistent with our previous report, which showed that reduced Pt species were more active in HCHO oxidation at room temperature than oxidized Pt species.^15^ The magnitude of reduction of the Pt species in the Pt/TiO_2_ catalysts varied, even though the catalysts were exposed to the same pretreatment conditions. In this study, the properties of the TiO_2_ supports varied substantially. Panagiotopoulou *et al.* mentioned that the activity of Pt/TiO_2_ catalysts during the water-gas shift (WGS) reaction was affected by the size of the TiO_2_ primary crystallite.^29^ Kim *et al.* also reported various activities for Pt/TiO_2_ catalysts in the reverse WGS reaction, with their catalytic nature depending on the properties of the TiO_2_ support. These studies suggested that the SMSI effect depended on the properties of TiO_2_ in the Pt/TiO_2_ catalyst.^21–23^ The results presented in Fig. 5 and 7 should, therefore, be related to TiO_2_ properties in the Pt/TiO_2_ catalyst. The relationships between reduced Pt species and TiO_2_ crystallite size in the Pt/TiO_2_ catalysts after pretreatment were investigated (Fig. 7(B)). In this analysis, the TiO_2_ crystallite sizes after catalyst preparation were evaluated, due to differences between the crystallite sizes in raw TiO_2_ and generated Pt/TiO_2_ catalysts.^22^ The relationship between both factors is also strongly correlated (Fig. 7(B)). Based on this result, and literature on the SMSI effect, smaller TiO_2_ crystallites could result in larger Pt/TiO_2_ interfaces, leading to an increase in the amount of oxygen vacancies, or Pt–O_v_–Ti^3+^ sites (Pt–Ti interfacial sites).^18,22,30^ The origin of differences in the activation energy required for the formation of Pt–O_v_–Ti^3+^ sites should be discussed. In general, noble metals, such as Ru, Pd, and Pt are not expected to interact strongly with TiO_2_.^31^ Dispersed noble-metal crystallites are well known to interact with partially reduced TiO_2_ supports, so smaller TiO_2_ supports exhibit better redox properties, which can also affect SMSI.

However, Huang and Leung reported that the effect of support reducibility or type of support on HCHO oxidation at room temperature was not important.^18^ They used CeO_2_, Al_2_O_3_, ZrO_2_, MgO, and TiO_2_ as supports, but support reducibility only weakly influenced catalyst activity. This result differs slightly from those presented in this study, which could be because the catalyst TOFs were calculated from activity obtained under low SV conditions. As discussed above, low SV resulted in a low performance level, making it difficult to determine the effect of support. In Fig. 4, the shift in the Pt 4f spectra was lower than that previously reported.^18^ Similarly, the shift in O 1s in Fig. 5 was slight. Nevertheless, the results of this study suggested that catalytic activity was dependent on support reducibility. All supports used in this study were TiO_2_, and their pretreatment conditions and methods were the same. The only differences were in the crystallite size. The activity tests were performed under extremely high SV conditions, therefore, support reducibility had a distinct effect on HCHO oxidation.

Zhang *et al.* reported a reaction model using *in situ* FTIR and argued that differences in catalytic activity varies with certain intermediate products, such as formate.^12,20^ Furthermore, formate was converted to CO when inert helium gas was injected. Kim *et al.* also reported that HCHO was adsorbed into formate and/or CO after formaldehyde flowed onto the catalyst surface, even in the absence of oxygen.^15^ The literature shows that formaldehyde was thought to convert into formate or CO species by a catalyst with active oxygen; therefore a discussion of the nature of this active oxygen would be worthwhile. If active oxygen is limited to existing on the catalyst, catalytic activity will decrease with time on stream. In contrast, if active oxygen can be recovered by atmospheric oxygen, catalytic activity will be stable with time on stream. Many previous studies on HCHO oxidation at room temperature over Pt/TiO_2_ catalysts showed no decrease in catalytic performance with time on stream during activity tests, therefore, active oxygen in Pt/TiO_2_ catalysts was thought to be recovered by atmospheric oxygen, with this phenomenon proceeding at room temperature.^16–18^

Until now, key factors affecting the activity of Pt/TiO_2_ catalysts in HCHO oxidation at room temperature were reported to be the valence of the active metal, and the amount of chemisorbed oxygen, however, their relationship is still unclear. To investigate the correlation between these two factors, an O_2_-reoxidation experiment was performed. When the TCD signal for all catalysts reached a steady state at room temperature, the consumed oxygen peaks were measured by feeding the catalyst with 1.5% O_2_/Ar gas.

**Fig. 8:**
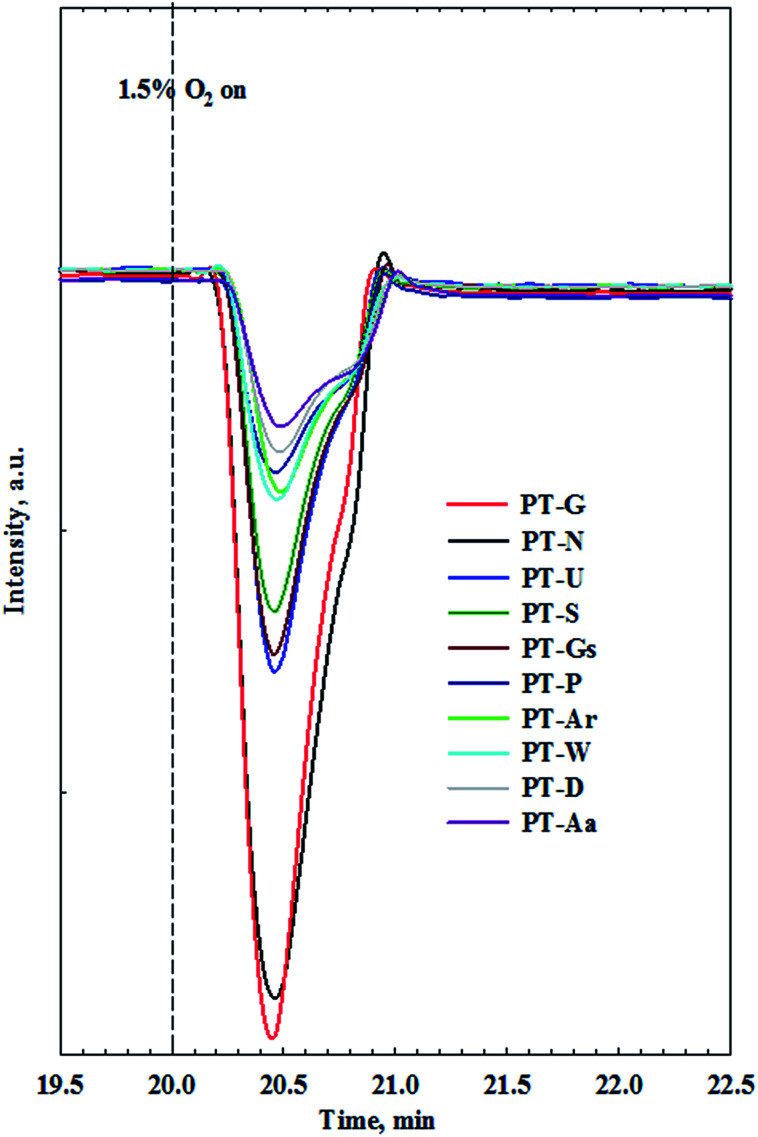
Response profiles of Pt/TiO_2_ catalysts in O_2_-reoxidation experiments.

Fig. 8 shows the consumed oxygen peaks over Pt/TiO_2_ catalysts. Oxygen was rapidly consumed after injection into the catalyst, and this effect appeared after oxygen adsorption onto the catalyst. Such oxygen consumption suggests that atmospheric oxygen can be adsorbed onto the Pt/TiO_2_ catalysts. Oxygen species adsorbed from the atmosphere can be used as active oxygen, which can participate in the conversion of HCHO into formate or CO. Interestingly, the amount of consumption differed among the different TiO_2_ supports. To examine the relationship between catalytic activity and O_2_ consumption rate, the area of oxygen consumed by each catalyst was compared with their catalytic activities.

**Fig. 9:**
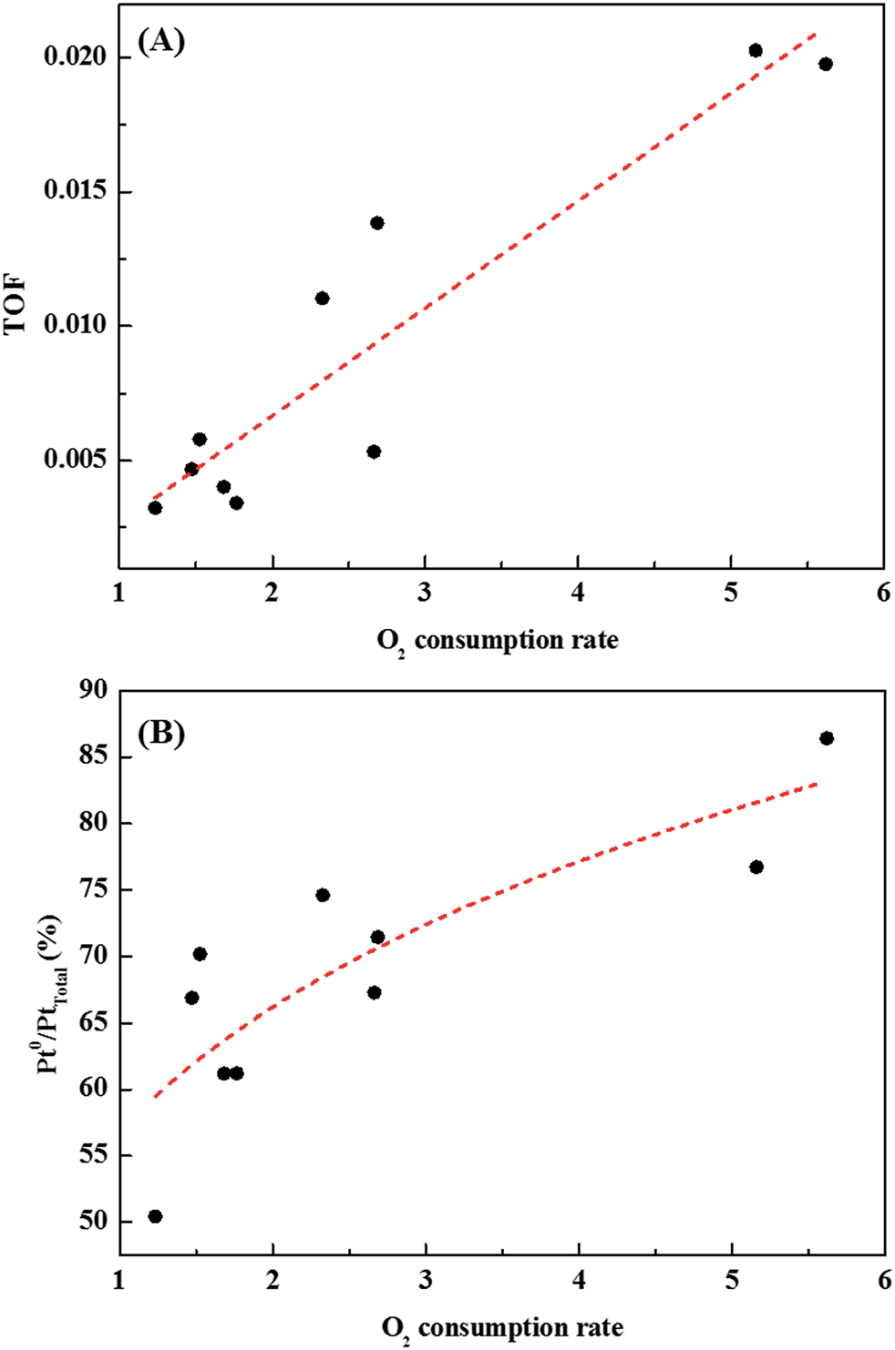
Correlations between (A) catalytic activity and catalyst O_2_ consumption rate, and (B) area of oxygen consumed by each catalyst and ratio of metallic Pt/Pt_total_ present in the catalyst.

Catalysts with excellent activity had good oxygen accepting abilities, while catalysts with poor activities had poor oxygen accepting abilities (Fig. 9(A)). In our previous study, we reported CO oxidation at room temperature over a reduced Pt/TiO_2_ catalyst, which had a high activity level and was rich in Pt^2+^ and Pt^0^ species, showing an excellent oxygen accepting ability, while the oxygen accepting ability of the calcined catalyst, which had a lower activity and was rich in Pt^4+^ species, was poor.^31^ This suggested that the adsorption of oxygen depended on the oxidation state of the Pt species. The strong correlation between the amount of oxygen consumed for each catalyst and the ratio of metallic Pt/Pt_total_ in each catalyst is shown in Fig. 9(B). As the area of oxygen consumed increased, the ratio of metallic Pt/Pt_total_ on the catalyst increased linearly. Catalysts with high metallic Pt/Pt_total_ ratios were thought to have more sites for accepting atmospheric oxygen due to them having more oxygen vacancy sites on the catalyst surface. The reduced catalyst was, therefore, thought to be able to adsorb atmospheric oxygen, which could then play a role as active oxygen, resulting in a continuous HCHO oxidation cycle at room temperature.

### 2.3 Effect of chemisorbed oxygen species on HCHO reaction over Pt/TiO_2_ catalyst and HCHO reaction mechanism

**Fig. 10:**
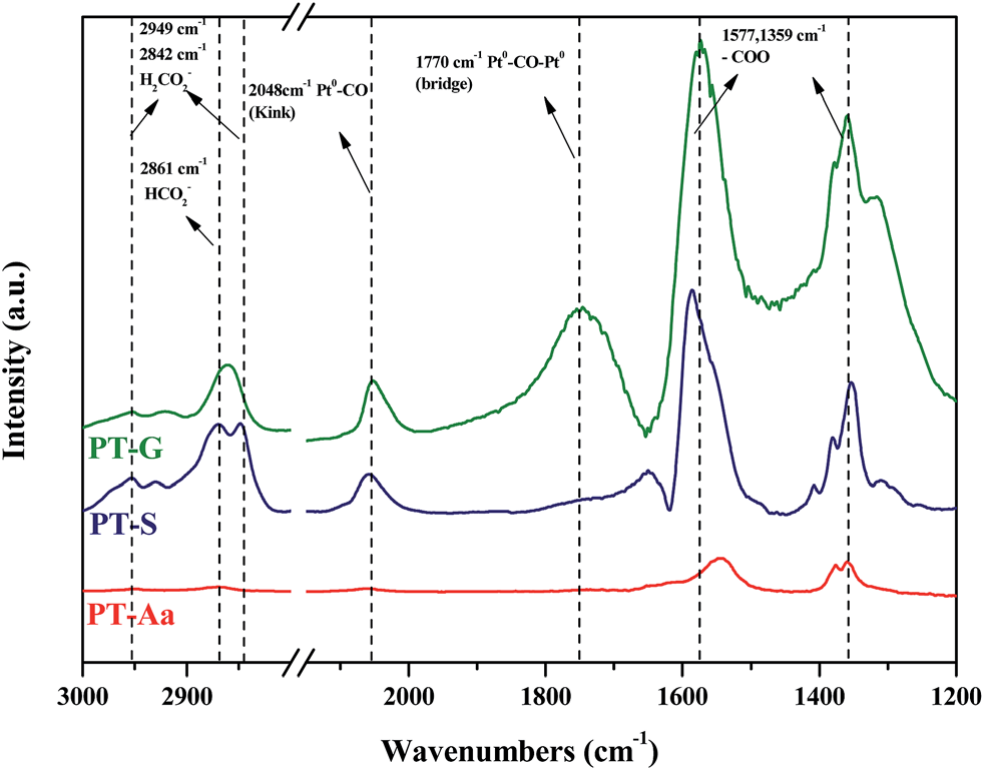
Dynamic changes in *in situ* FT-IR spectra of the Pt/TiO_2_ (G), Pt/TiO_2_ (S), Pt/TiO_2_ (Aa) catalysts as a function of time in a gas flow of HCHO mixed with N_2_ at room temperature. Inlet HCHO, 20 ppm.

DRIFTS studies of HCHO adsorption and the effect of chemisorbed oxygen species on adsorbed HCHO were investigated using Pt/TiO_2_–S, Pt/TiO_2_–G, and Pt/TiO_2_–Ar catalysts. Fig. 10 shows changes in the peaks upon injection of HCHO (20 ppm) into the reactor containing the Pt/TiO_2_ catalysts at room temperature.

As shown in Fig. 10, after HCHO adsorption on the Pt/TiO_2_ catalysts, positive bands were noticeable at 2861, 2842, 2048, 1770, 1577, 1359 cm^−1^. The band at 2861 cm^−1^ represented the formate species (HCOO), while the bands at 2842 cm^−1^ represented the formic acid species (HCOOH).^20,32–36^ The band at 2048 cm^−1^ was assigned to linearly adsorbed CO on metallic Pt (kink) sites, while the band at 1770 cm^−1^ was assigned to CO bridged between two Pt metal particles.^20,37^ Peaks at 1570 and 1360 cm^−1^ indicated the presence of formate, while negative bands were observed at 1635 cm^−1^ and broad bands were observed in the range 3600–3200 cm^−1^.^36^ Generally, the broader peaks at 3200–3600 cm^−1^ and strong peaks at 1635 cm^−1^ were assigned to the O–H stretching vibrations of water, –OH groups, and hydrated species on the catalyst surface.^34,36^ Nie *et al.* and Chen *et al.* reported that HCHO adsorbed on the hydroxyl group over the catalyst surface.^13,38^ Therefore, the 1635 cm^−1^ negative peak was due to the adsorption of HCHO on –OH groups.

The same peaks were observed in all catalysts. These results suggested that the same intermediate product was formed on each catalyst surface. Peak intensities differed with each type of TiO_2_, however. In this study, Pt/TiO_2_–G, which showed excellent O_2_ consumption in Section 2.2, had large formate peak area at 1570 and 1360 cm^−1^, confirming that a large amount of HCHO was adsorbed and converted to formate by HCHO injection. The peaks at 2048 and 1770 cm^−1^ were also large, resulting from formation of the CO intermediate product. In contrast, the Pt/TiO_2_–Ar catalyst showed low O_2_ consumption after HCHO injection, with small peaks at 1570 and 1360 cm^−1^, therefore, the amount of formate was smaller than that of the Pt/TiO_2_–G catalyst, and the CO adsorption peaks at 2048 and 1770 cm^−1^ were also small. It was consistent with our previous study, which reported that surface CO species were the main intermediates in HCHO oxidation.^15^ Zhang and He also reported that differences in catalytic activities were observed because formate species on the catalyst surface did not easily convert into CO on any of the active metals (Rh, Pd, and Au).^20^ Therefore, catalyst O_2_ consumption, as shown in Fig. 8, was involved in the formation of the formate intermediate from HCHO adsorption. The reaction mechanism will be explained in detail using DRIFTS analysis.

**Fig. 11:**
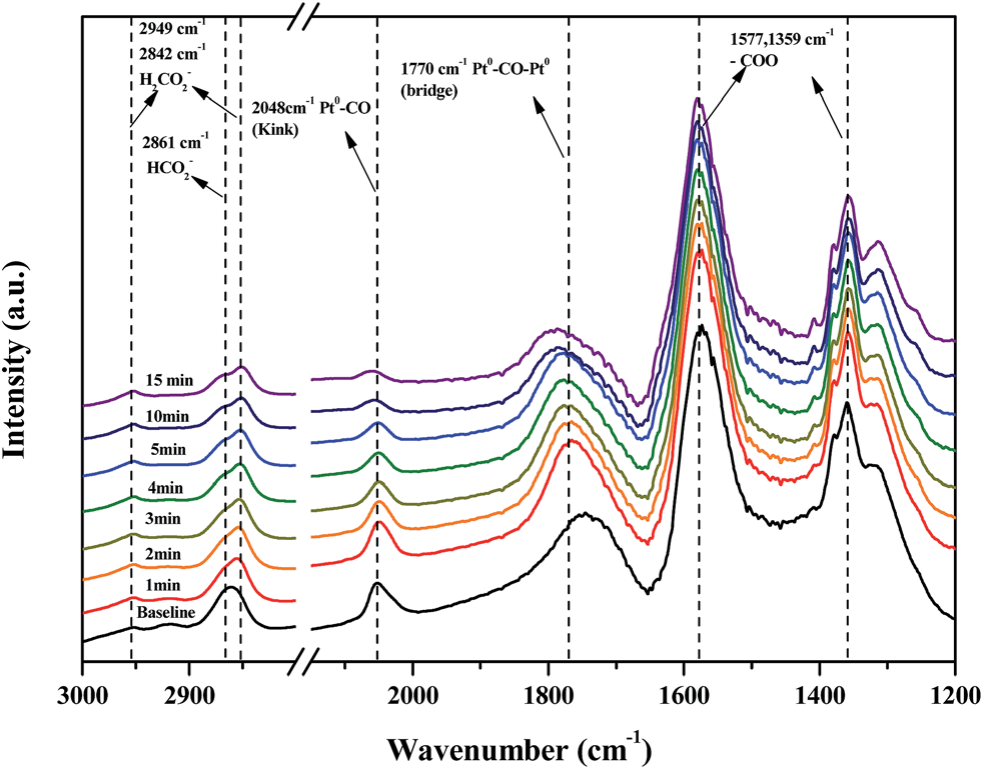
Dynamic changes in *in situ* FT-IR spectra of the O_2_ reaction after HCHO adsorbance over Pt/TiO_2_ (G) catalyst as a function of time in a gas flow of 21% O_2_/N_2_ at room temperature.

Fig. 11 shows changes in the peaks upon injecting O_2_ and N_2_ gas into the Pt/TiO_2_–G catalysts for 30 min to investigate changes in the adsorption characteristics of the HCHO species adsorbed on the catalyst surface. As a result, under oxygen injection conditions, the formate peaks at 2861, 2842, 1577 and 1359 cm^−1^ showed no significant fluctuations, meaning that formate species did not react with oxygen in the air and that no oxygen in the air was used in the conversion of formate species to CO. In contrast, the size of peaks at 2048 and 1770 cm^−1^ for CO adsorbed to metallic Pt were reduced, meaning that CO adsorbed to metallic Pt reacted with oxygen in air, followed by conversion to CO_2_ and desorption. This explains the results in Fig. 7(A). Bourane *et al.* examined the adsorption and desorption of CO on the surface of a Pt/Al_2_O_3_ catalyst *via* FT-IR analysis, reporting that the linear CO species was adsorbed on either metallic Pt or Pt^2+^, while the bridged CO species was adsorbed between two metallic species.^39^ Representative CO adsorption peaks were observed in this study at 2048 (linear CO on metallic Pt) and 1770 cm^−1^ (bridged CO on metallic Pt), however, the CO adsorption peak corresponding to Pt^2+^ was not observed. Therefore, conversion of HCHO and CO absorption occurred only at metallic Pt sites.

**Fig. 12:**
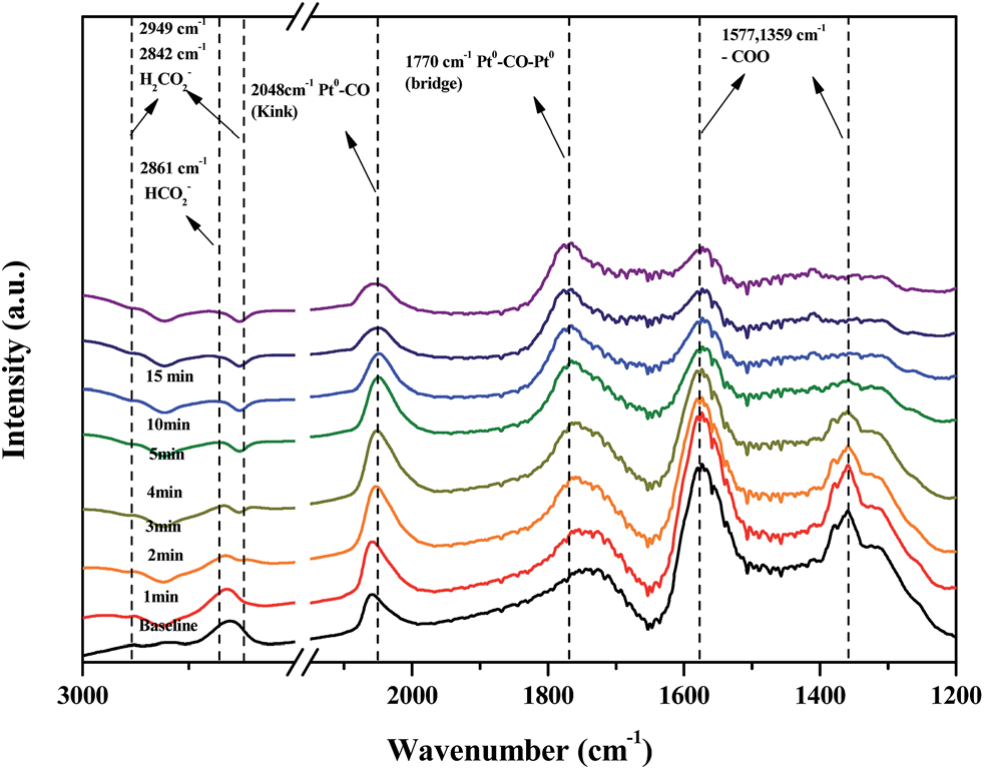
Dynamic changes in *in situ* FT-IR spectra of the H_2_O reaction after HCHO adsorbance over Pt/TiO_2_ (G) catalyst as a function of time in a gas flow of H_2_O/N_2_ at room temperature. Inlet H_2_O = 55% relative humidity.

Fig. 12 shows changes in peaks upon injecting H_2_O and N_2_ gas into Pt/TiO_2_–G catalysts for 30 min to investigate the changes in adsorption characteristics of the HCHO species on the catalyst surface. The peak at 2842 cm^−1^, originating from HCOOH species after injecting moisture, decreased rapidly, with no peak observed after 3 min. After injecting moisture, the formate peaks at 2661, 1577, and 1359 cm^−1^ also decreased sharply. No peaks were observed at 2861 and 1359 cm^−1^ after 4 min, while the size of the peak at 1577 cm^−1^ reduced. In contrast, the peak at 2048 cm^−1^, originating from CO linearly adsorbed on metallic Pt sites, continued to increase up to 3 min but decreased after 3 min. The bridged CO peak (1770 cm^−1^) increased up to 4 min after injecting moisture. HCOOH species and formate species had formed on the catalyst surface after the injection of HCHO, which were converted to CO, which then desorbed as CO_2_ after reaction.

**Scheme 1:**
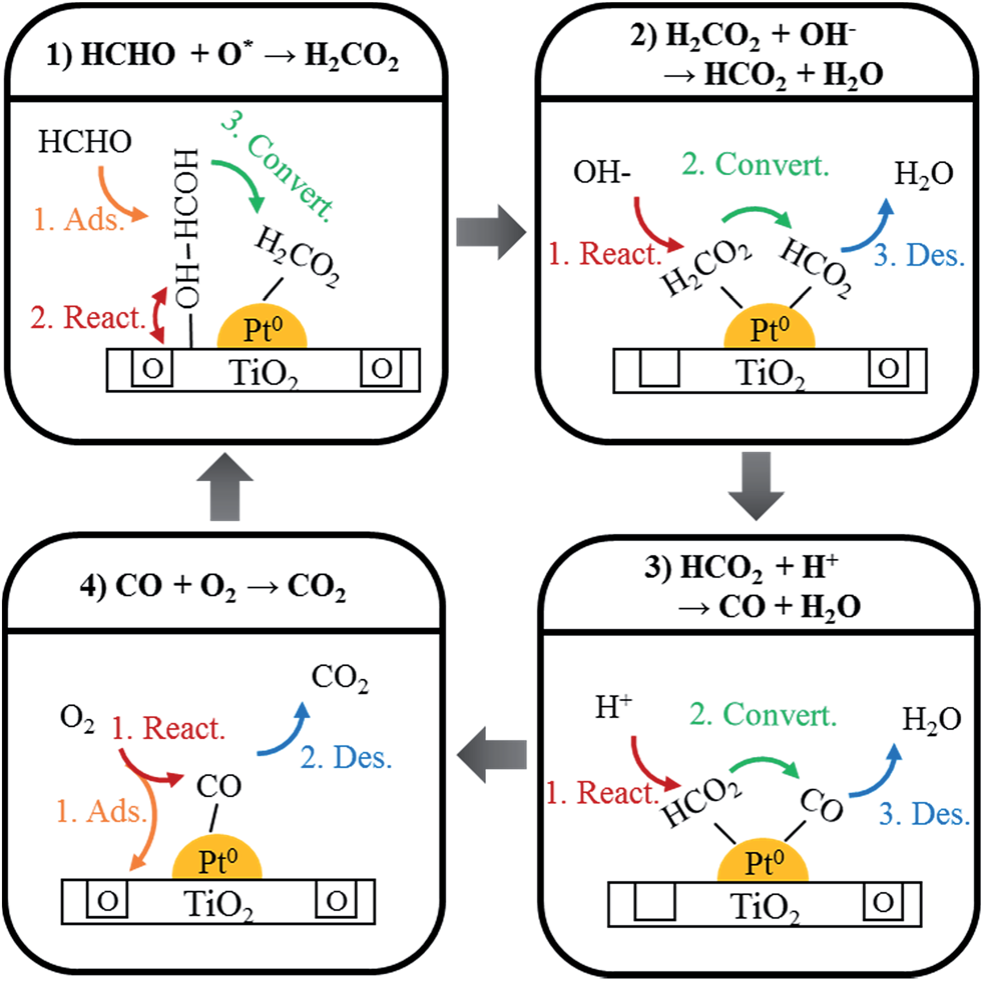
Schematic model of HCHO oxidation over Pt/TiO_2_.

Using DRIFTS analysis, the room temperature HCHO oxidation mechanism was identified, as shown in Scheme 1. First, HCHO was converted and absorbed as HCOOH by a reaction with oxygen at the catalyst surface (eqn (1))

1 HCHO + O^*^_surf._ → HCOOH

This HCOOH was converted to adsorbed CO (eqn (2) and (3)).

2 HCOOH + OH^−^ → HCOO + H_2_O

3 HCOO + H^+^ → CO + H_2_O

Adsorbed CO species were converted into CO_2_ by the reaction with oxygen in air, and desorbed (eqn (4)).

4 2CO + O_2 gas_ → CO_2_

**Fig. 13:**
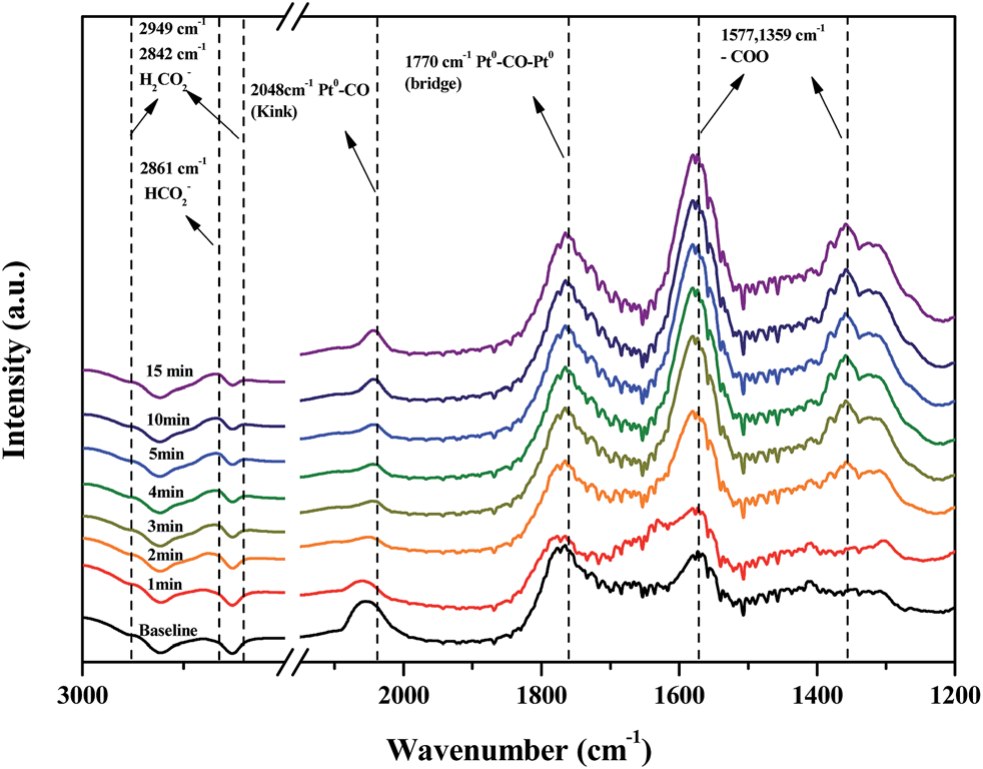
Dynamic changes in *in situ* FT-IR spectra of HCHO + O_2_ + H_2_O reaction over Pt/TiO_2_ (G) catalyst as a function of time in a gas flow of HCHO/O_2_/H_2_O/N_2_ at room temperature. Inlet HCHO, 20 ppm; inlet H_2_O = 55% relative humidity.

Fig. 13 shows the changes in peaks upon injecting HCHO/H_2_O/O_2_/N_2_ mixed gas into the Pt/TiO_2_–G catalysts for 30 min to investigate the changes in adsorption characteristics of the HCHO species on the catalyst surface. Formate peaks at 2861, 1577, and 1359 cm^−1^ were observed after gas injection and CO species adsorbed on metallic Pt were observed at 2048 and 1770 cm^−1^. The HCOOH adsorption peak at 2842 cm^−1^ was not observed, as shown in Fig. 10. This lack of adsorption peak for HCOOH in the HCHO reaction by DRIFTS analysis suggested that the conversion of adsorbed HCOOH to HCOO and CO species was rapid (eqn (2) and (3)). The peak at 2048 cm^−1^, generated by CO linearly adsorbed on metallic Pt sites, grew slowly after a certain response time. This result (eqn (4)) indicated a slower response speed (eqn (2) and (3)), therefore, two catalyst factors were identified that affected the response speed in the room temperature oxidation of HCHO. First, adsorbed surface oxygen in the catalyst formed HCOOH intermediate species on the catalyst surface (eqn (1)). This result coincided with excellent HCHO reaction activity for catalysts, for which the HCOOH peak was large. Surface oxygen was replenished by oxygen in the air, therefore, the same result of excellent HCHO reaction activity was achieved by catalysts with excellent O_2_-consumption. The second factor was the Pt valence state and Pt dispersion, which determined the response speed (eqn (4)). The room temperature oxidation response of HCHO was confirmed in Fig. 10, as it was converted and adsorbed to metallic Pt as CO species and responded to O_2_. Therefore, an increase in the metallic Pt ratio, which was the active site, and high catalyst dispersion, increased the final response speed (eqn (4)) to show excellent activity.

## 3 Experimental

### 3.1 Catalyst preparation

A TiO_2_-supported Pt catalyst was prepared with the wet impregnation method, using various commercially available TiO_2_ supports. Ten types of commercial TiO_2_ supports were purchased from Millennium Chemical (types G, Gs, and D), Ishihara (type S), Hombikat (type U), Sigma-Aldrich (types Aa and Ar), Degussa (type P), Wako (type W), and Nano (type N). The content of TiO_2_ platinum was calculated by weight ratio, and then platinum chloride (PtCl_4_; Aldrich Co.) was dissolved in distilled water at 333 K. The calculated amount of target TiO_2_ was added to the solution. The resultant slurry was stirred for over 1 h, and then evaporated at 343 K using a evaporator (N-N series, Eyela Co.). The product was dried at 376 K overnight. To remove residual chlorine, the sample was reduced in a tubular furnace at 673 K under 30% hydrogen gas. Finally, the sample was calcined at 673 K under an air-pressure atmosphere, and then reduced using hydrogen gas at 873 K for 1 h to create the final catalyst.^14^

### 3.2 Test of catalytic activity

The fixed bed reactor had a part for gas injection part, an oxidation column, a reactor, and a part for analyzing the reaction gas. An impinger containing paraformaldehyde was placed in a constant-temperature tank, maintained at 295 K, and HCHO was put into the reactor by vaporization of the paraformaldehyde, supplying a constant amount of carrier gas to the impinger.^14,15^ The temperature of the impinger was kept so that the HCHO concentration in the generated gas was 24 ppm. Moisture was supplied by injecting moist air using a bubbler. To maintain a constant supply of moist air, water was circulated at a constant temperature (298 K) using a circulator, in the form of a double jacket, outside the bubbler. The reactor used a continuous flow fixed bed reactor with a quartz tube (inner diameter 8 mm). Reactor temperature was controlled by filling the outer reactor with water, with a K-type thermocouple installed to measure temperature.

To confirm initial and product concentrations, CO_2_ & CO were analyzed using a non-dispersive infrared (NDIR) analyzer (ZKJ-2, Fuji Electric Co.). To measure the concentration of CO_2_ converted from injected HCHO, a catalytic oxidation reactor was used. 1 mole of CO_2_ was produced by 1 oxidized mole of HCHO. The oxidation tower operated at 300 °C, which can completely oxidize the injected HCHO to CO_2_, confirmed by measuring unreacted HCHO at the tail end of the fixed bed reactor using a total hydrocarbon (THC) analyzer (Thermo 55 °C) and detector tube (CO, HCHO, MeOH, HCHO, and HCOO). Gases other than CO_2_ were not detected.

The inlet CO_2_ concentration was measured using a NDIR analyzer after the paraformaldehyde was vaporized by the oxidation catalyst tower. The outlet concentration of CO_2_ was measured after passing through each filled Pt/TiO_2_ catalyst bed reactor at 298 K. The outlet concentration was analyzed after gases were at a steady state.

### 3.3 Catalyst characterizations

The surface areas of the Pt/TiO_2_ catalysts were measured using an ASAP 2010C instrument (Micromeritics), and calculated by using the Brunauer–Emmett–Teller (BET) equation. Each sample was analyzed after being degassed under vacuum at 383 K for 3 h.

To observe the crystal structure of the Pt/TiO_2_ catalysts, X-ray diffraction (XRD) analysis was conducted. Cu Kα was used as the radiation source (*λ* = 0.1506 nm). The catalyst measurements were made in the 2theta range of 20–90°, at a scanning velocity of 4° min^−1^.

X-ray photoelectron spectroscopy (XPS) was performed using an ESCALAB 210 spectrometer (VG Scientific), with AlKα monochromate (1486.6 eV) as an excitation source. After the catalyst was dried for 24 hours at 373 K and the moisture was completely removed, the catalyst was analyzed without surface sputtering and etching to maintain the vacuum of the XPS apparatus at 10–12 mmHg. The bond energies and intensities of Pt, O, Ti, and C in the sample were analyzed with the wide scanning spectrum.

To evaluate the reoxidation abilities of the catalysts, we conducted an O_2_-reoxidation experiment. The filled catalyst was activated for 10 minutes at 393 K with 50 cm^3^ min^−1^ Ar. The catalyst was cooled to room temperature (306 K), the thermal conduction detector (TCD) signal was stabilized, and a continuous supply of 1.5% O_2_/N_2_ was provided to obtain O_2_ concentration TCD.^19^

Field emission-transmission electron microscopy (FE-TEM) were performed on a JEM-2100F microscope (JEOL Co.) operated at 200 kV. The FE-TEM sample was prepared by suspending the sonicated catalyst powder in ethanol and this suspension was dropped into a Cu grid.

The *in situ* DRIFTS analysis used in this study was carried out with a FT-IR spectrometer (Nicolet IS 10, Thermo Fisher, USA), and a diffuse reflectance (DR) 400 accessory was used for solid reflectance analysis. The spectrum contained 30 cumulative scans with a resolution (4 cm^−1^) by a mercury-cadmium-telluride (MCT) detector. To exclude the influence of moisture and impurities, samples were pre-processed under Ar at 423 K for 1 h, and then maintained in a vacuum state using a vacuum pump. HCHO was injected by using vaporized paraformaldehyde, obtained by injecting N_2_-containing HCHO vapor through the paraformaldehyde, and N_2_ gas to the impinger.

CO chemisorption analysis was performed at 298 K to analyze the crystal size to aid dispersion of the catalyst. The catalyst sample, which was pretreated in a hydrogen flow for 30 minutes at 573 K, was cooled down to 298 K and then saturated with the injection of a CO pulse.

Reaction rates were obtained using the below equation:
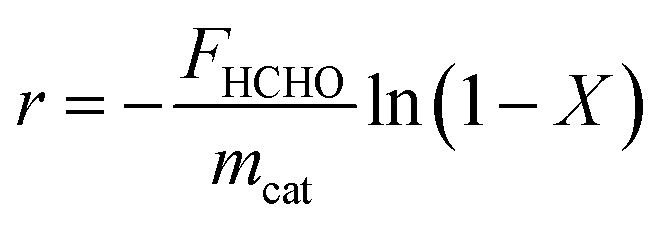
where *r* is the HCHO reaction rate (mol s^−1^ g_cat_^−1^), *F*_HCHO_ is the total HCHO flow rate (mol s^−1^), *m*_cat_ is the weight of the catalyst (g), and *X* is the HCHO conversion.^40,41^ These results, along with metal dispersion measurements, were used to calculate HCHO turnover frequency (TOF), obtained as amount of moles of HCHO converted per surface active metal atom per second (s^−1^):
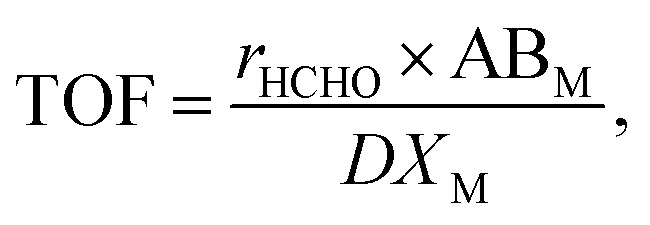
where AB_M_ represents the atomic weight of active metal M, *X*_M_ is the active metal content (g_met_ g_cat_^−1^), and *D* is the active metal dispersion.^8^

## 4 Conclusions

In this study, different Pt/TiO_2_ catalysts prepared by various TiO_2_ showed different catalytic activities. XPS analysis demonstrated that the catalytic activities were related to Pt valence states and chemisorbed oxygen species on the Pt/TiO_2_ catalysts, and these characteristics were dependent on the type of TiO_2_. O_2_ reoxidation experiments confirmed that the areas of oxygen consumed and metallic Pt species on the catalyst surface were strongly correlated. DRIFTS analysis showed that the chemisorbed oxygen species were involved in the formation of formate species. These formate species reacted with moisture, converting to linear CO species on metallic Pt sites. Finally, the linear CO species reacted with gaseous oxygen to desorb as CO_2_, completing the mechanism.

## Conflicts of interest

There are no conflicts to declare.

## Supplementary Material
